# High-Temperature Core Flood Investigation of Nanocellulose as a Green Additive for Enhanced Oil Recovery

**DOI:** 10.3390/nano9050665

**Published:** 2019-04-27

**Authors:** Reidun C. Aadland, Trygve D. Jakobsen, Ellinor B. Heggset, Haili Long-Sanouiller, Sébastien Simon, Kristofer G. Paso, Kristin Syverud, Ole Torsæter

**Affiliations:** 1 Department of Geoscience and Petroleum, PoreLab Center of Excellence, Norwegian University of Science and Technology (NTNU), N-7491 Trondheim, Norway; haili.long-sanouiller@ntnu.no (H.L.-S.); ole.torsater@ntnu.no (O.T.); 2 Department of Chemical Engineering, Norwegian University of Science and Technology (NTNU), N-7491 Trondheim, Norway; sebastien.c.simon@ntnu.no (S.S.); kristofer.g.paso@ntnu.no (K.G.P.); kristin.syverud@rise-pfi.no (K.S.); 3 RISE PFI, N-7491 Trondheim, Norway; ellinor.heggset@rise-pfi.no

**Keywords:** enhanced oil recovery, nanocellulose, petroleum, cellulose nanocrystals, tertiary recovery, crude oil, nanoparticle, CNC, core flood, high temperature, heat aging, rheology modification

## Abstract

Recent studies have discovered a substantial viscosity increase of aqueous cellulose nanocrystal (CNC) dispersions upon heat aging at temperatures above 90 °C. This distinct change in material properties at very low concentrations in water has been proposed as an active mechanism for enhanced oil recovery (EOR), as highly viscous fluid may improve macroscopic sweep efficiencies and mitigate viscous fingering. A high-temperature (120 °C) core flood experiment was carried out with 1 wt. % CNC in low salinity brine on a 60 cm-long sandstone core outcrop initially saturated with crude oil. A flow rate corresponding to 24 h per pore volume was applied to ensure sufficient viscosification time within the porous media. The total oil recovery was 62.2%, including 1.2% oil being produced during CNC flooding. Creation of local log-jams inside the porous media appears to be the dominant mechanism for additional oil recovery during nano flooding. The permeability was reduced by 89.5% during the core flood, and a thin layer of nanocellulose film was observed at the inlet of the core plug. CNC fluid and core flood effluent was analyzed using atomic force microscopy (AFM), particle size analysis, and shear rheology. The effluent was largely unchanged after passing through the core over a time period of 24 h. After the core outcrop was rinsed, a micro computed tomography (micro-CT) was used to examine heterogeneity of the core. The core was found to be homogeneous.

## 1. Introduction

The average oil recovery factor (RF) on the Norwegian Continental Shelf (NCS) is 47% and on a global basis the average recovery factor remains less than 40% [1]. In addition, oil production rates at existing fields are declining and the frequency of new field discoveries has diminished substantially in the last few decades [2]. Thus, it is essential to target the residual oil remaining in petroleum reservoirs and achieve higher recovery factors from existing fields. Deployment of new and innovative production technologies is necessary to achieve this goal. One of the technologies is called enhanced oil recovery (EOR) and includes any technique where fluids/material that are not normally present in a reservoir is injected into it. The three primary categories of EOR include: thermal, chemical and miscible or solvent injection [3]. Thermal recovery is typically utilized in heavy oil reservoirs. The method applies heat to reduce oil viscosity, facilitating mobilization of oil towards the production well. Chemical methods fall into three different groups: polymer flooding, surfactant-polymer (SP) flooding and alkaline-surfactant-polymer (ASP) flooding. The main idea is to add chemicals to the water flooding stage to either increase the mobility ratio (polymer), reduce the interfacial tension (surfactant), or create micro-emulsions (alkali). Miscible or solvent injection involves injection of a mutually miscible low viscosity solvent to mobilize residual oil trapped in the reservoir rock [4,5]. Chemical flooding with polymer additives is considered the most promising EOR method [6] for implementation on the Norwegian continental shelf.

**Figure 1:**
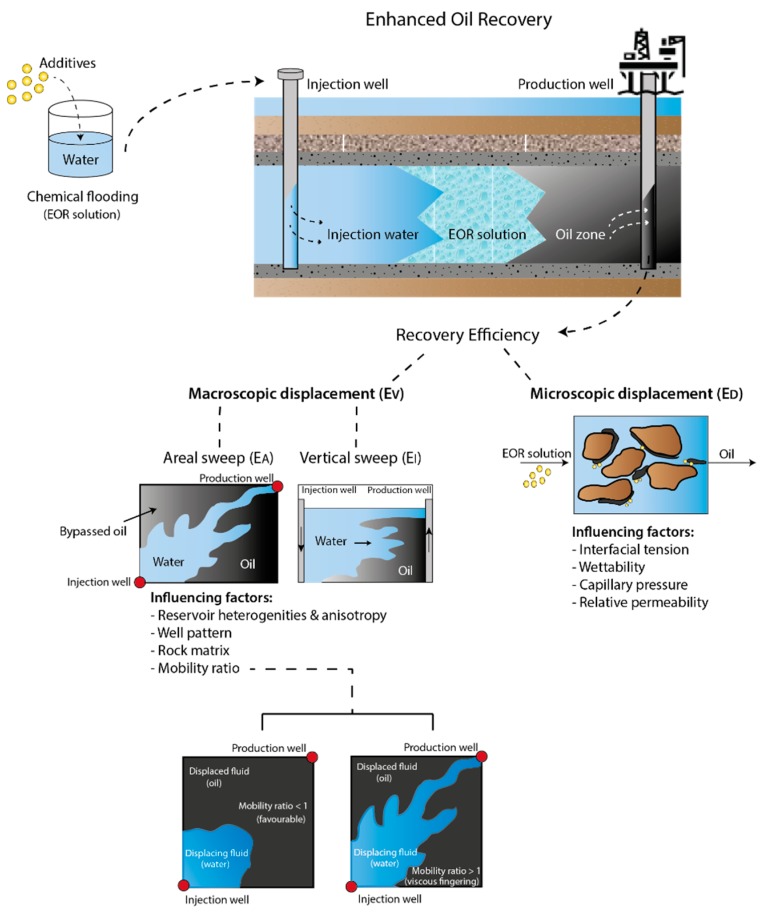
Schematic illustration of enhanced oil recovery and the overall recovery efficiency.

The main objective of EOR methods is to increase the overall recovery efficiency (*E*), which may be quantified as the product of the macroscopic or volumetric displacement efficiency (EV) and the microscopic displacement efficiency (ED), as shown in Equation (1) [7]. A schematic illustration of the recovery process and specific definitions can be found in Figure 1.

(1) $$E=\;E_{V}E_{D}$$

Microscopic displacement efficiency refers to trapping of residual oil at the pore level (Figure 1). The term ED quantifies how well displacing fluid mobilizes residual oil upon contact. At the pore level, various factors influence oil displacement, such as interfacial tension, wettability, capillary pressure and relative permeability. For oil/brine systems, interfacial tension values are typically in the order of 20–30 mN/m [7,8]. However, by adding tailored chemicals to the water phase, it is possible to reduce the interfacial tension by several orders of magnitude [7]. A lower interfacial tension allows extraction of more oil, as the oil droplets will become dislodged from the pore throats.

The macroscopic displacement efficiency term may be quantified as the product of the areal (EA) and vertical (EI) sweep efficiencies, where EA denotes the horizontal area that can be swept and EI denotes the cross-sectional area that can be invaded (Figure 1). Overall, the EV term describes how well the injected fluid has traversed the reservoir, i.e., which fraction of the reservoir the displacing fluid has been able to contact. Several factors affect the macroscopic sweep efficiency, such as mobility ratio, reservoir heterogeneities and anisotropy, well pattern, and the type of rock matrix imbibed with oil.

The mobility ratio (M) is a measure of how easy one fluid moves through the porous media relative to another fluid, and is given in Equation (2) [9].

(2) $$M=\frac{mobility\;of\;displacing\;fluid}{mobility\;of\;displaced\;fluid}=\;\frac{k_{rw}\mu_{o}}{k_{ro}\mu_{w}}$$

where *k_rw_* and *k_ro_* denote the effective permeabilities of the displacing fluid (e.g., water) and displaced fluid (oil), respectively, and µ*_w_* and µ*_o_* denote the corresponding viscosities, respectively. The efficiency of the volumetric sweep increases as the mobility ratio decreases. Thus, a favorable mobility ratio is obtained if *M* < 1. If *M* > 1 the mobility ratio is considered unfavorable, because the displacing fluid (water) flows with less viscous resistance than the displaced fluid (oil). This imbalance will result in the displacement fluid bypassing oil pockets and oil-filled pores because of viscous fingering and an unstable fluid front (Figure 1). Viscous fingering occurs as a result of the viscosity contrast and effectively reduces the oil recovery efficiency [7,9].

The displacing fluid should ideally have a high viscosity in order to be able to sweep the reservoir more effectively. A favorable mobility ratio can thus be achieved by altering the parameters in Equation (2). For a given reservoir with a specified oil, the parameter that can be controlled is the viscosity of the injection fluid. However, it is important that the fluid has a sufficiently low initial viscosity to make it easy to pump into the reservoir. The optimal solution is, therefore, to have a low viscosity during injection, and then as the fluid enters the reservoir it would increase in viscosity with time.

A common technique for increasing the displacement fluid viscosity is to add polymers to the injection water. Shiran et al. [10] performed laboratory experiments in which polymer flooding was introduced as a tertiary recovery technique (after water flooding). To obtain a favorable mobility ratio, a diluted crude oil with a viscosity of 2.4 cP was used. Two polymer solutions with viscosities of 2.6 and 2.2 cP were tested. Three Bentheimer sandstone cores were used with different wettabilities, and all cores had a permeability value of approximately 2 D. The quantity of additional oil recovered after water flooding ranged from 3.2%–12.9%. The low percentage of 3.2% was achieved when the core plug was preferentially water-wet, while the highest percentage of 12.9% was achieved when the core plug was preferentially oil-wet. A lower recovery efficiency and higher residual oil is generally observed in strongly water-wet porous media. The reason for this is that oil may remain in larger pores where it could be snapped off, or a water film may disconnect a continuous mass of oil. In both scenarios the oil becomes trapped in the pore structure of the porous media [10].

Two main polymer types are currently in use commercially; xanthan gum and partially hydrolyzed polyacrylamide (HPAM) [11]. Both of these serve to reduce the mobility of water by increasing the viscosity. However, both polymers have substantial performance limitations with respect to EOR. Xanthan gum can plug formations, is prone to microbial degradation, and exhibits poor thermal stability [12]. HPAM, on the other hand, is susceptible to numerous chemical, thermal and mechanical degradation mechanisms. Thus, the viscosifying capability of HPAM will gradually diminish as it migrates through porous media [7,12]. As many of the polymers used today are considered toxic and detrimental to the environment, it is of importance to develop stable and green alternatives to this EOR technique. This has been a focus area in research over the last decade, where many different types of green alternatives have been tested [13,14,15,16,17,18,19,20,21,22].

Nanocellulose is introduced as another green and environmental friendly alternative to existing EOR polymers. Nanocellulose is nanoscaled particles composed of the biopolymer cellulose, and is an abundant and renewable natural resource. Cellulose is the structural component of plant cells [23], and the two most common cellulosic sources are wood and cotton. According to Klemm et al. [24] cellulose is almost an inexhaustible source of raw material and thereby perfectly able to supply the increasing demand for environmentally friendly and biocompatible products. A tree produces 10 g of cellulose per day and the total production of cellulose all over the world is estimated to be 1.3 × 10^10^ tons per year [23]. The majority of forest areas utilized for commercial wood conversion are managed based on the criterion of sustainable development, meaning that the annual growth of the forests is considerably greater than what is removed. In addition, since nanocellulose can be produced from unused (today) remains from paper production, no new consumption of trees may even be necessary. Thus, extensive use of nanocellulose should not have a large effect on the environment. Another positive attribute with nanocellulose in terms of offshore EOR applications is that it can be discharged into the ocean. Nanocellulose would agglomerate in high salinity water and degrade.

Disintegration of wood fibers into cellulose nanofibrils (CNFs) was first described by Turbak et al. [25] and Herrick et al. [26]. Similar materials called cellulose nanocrystals (CNCs) were studied as early as 1949 [27] and described as rod-like cellulose micelles by Rånby and Ribi [28], and the current study focuses on CNC material. CNCs are produced from acid hydrolysis of cellulosic material, which dissolves amorphous sections, leaving crystalline, rod-like particles intact. Sonication or similar methods are often required to obtain dispersed particles [29]. The particles are typically 100 to 250 nm in length and 5 to 70 nm wide.

There does not exist much literature on nanocellulose for enhanced oil-recovery applications. However, preliminary experiments have investigated the potential of nanocelluloses as the EOR agent. For instance, retention is a commonly encountered problem when developing EOR polymers. Molnes et al. [19] investigated injectivity of nanocellulose through porous media, and found that the particles traversed the core. Nevertheless, there were indications that a portion of the CNC particles became trapped inside the pore matrix [19]. Aadland et al. [20] performed a more extensive retention study on CNCs in core plugs and sandpacks and observed that salinity had the largest effect on retention. The study concluded that CNCs dispersed in low-salinity water could have promising potential as EOR agents [20]. Jakobsen et al. [22] investigated interactions between anionic surfactants and nanocellulose and found that both surfactants and nanocellulose play a role in lowering interfacial tension between oil and water, and that the presence of surfactant increased the viscosity of the nanocellulose dispersions by effectively binding together nanocellulose particles.

Temperature conditions in petroleum reservoirs may be quite high (>100 °C), a potential EOR agent should, therefore, be thermally stable within the temperature limits of oil reservoir applications. Heggset et al. [30] performed a temperature stability study where nanocellulose exhibited improved thermal stability in comparison to guar gum and xanthan gum [30]. Molnes et al. [18] also performed a heat aging study on CNCs, where shear viscosity was measured after 24, 48 and 168 h of heating. The study demonstrated that shear viscosity increases drastically after only 24 h of aging at 120 °C. The results collectively demonstrate that a certain aging period is required under high temperature conditions to obtain a dramatic increase in shear viscosity for CNCs in low salinity water [18].

In a subsequent study by Molnes et al. [17] the injectivity of CNC dispersions at 60, 90 and 120 °C was investigated using core plugs. They observed no viscosity increase for any of the tests. A possible explanation for this was that the residence time of the CNCs in the matrix was insufficient for modification of the CNC structure. For their tested flow rate (4 pore volume (PV)/day), a CNC particle would take approximately six hours to traverse the core. The heat aging experiment revealed that the increase in viscosity was not observed before the dispersion had been aged for at least 20 h [18]. They also performed two oil recovery experiments using nanocellulose on Berea sandstone cores. CNCs in low salinity water was injected as a tertiary recovery technique at 60 °C and 90 °C and the incremental oil recovery was calculated. It was concluded that CNCs dispersed in low salinity water might have a certain potential for use in enhanced oil recovery. Additional oil produced attributable to nanocellulose was 3.4% for the test at 90 °C, while no significant EOR effect was observed for the test performed at 60 °C [17].

The current investigation is an extension of the heat aging experiments performed by Molnes et al. [17,18]. The potential of CNCs as an EOR agent was tested by performing a core flood experiment in low salinity water. The core flood was performed at 120 °C using a long core (approx. 60 cm) and low flow rate in order for the CNC solution to have a long residence time within the porous media. The goal was to reproduce the same viscosification effect that was revealed in the Molnes et al. [18] study, and to determine the amount of incremental oil produced after water flooding. Permeability measurements were also conducted during the core flood.

## 2. Materials

### 2.1. Porous Media

Bentheimer sandstone was used as the porous media. The core sample had a permeability of 2.0 Darcy and porosity of 21.6%. The pore volume (PV) was 141.8 mL. Length and diameter were 59.2 cm and 3.76 cm, respectively. The absolute permeability was measured using brine.

### 2.2. Brine

Experiments were performed using low salinity water, consisting of 0.1 wt. % sodium chloride (NaCl) mixed with de-ionized water (DIW). After mixing, it was filtered using a 0.45 μm Millipore filter under vacuum. Brine properties are listed in Table 1. 

### 2.3. Oil

During the saturation phase of the core, various types of oil were used and their properties are listed in Table 1. Isopar L (ExxonMobil Chemical Europe, Mechelen, Belgium) was applied first, then a mix of 20% isopar L and 80% high-viscous paraffin (HVP) oil (VWR International AS, Oslo, Norway). At the end, when the desired initial water saturation was reached, the 20/80% isopar L/HVP solution was replaced with stabilized and degassed crude oil. From the SARA (Saturates, Aromatic, Resin and Asphaltene) analysis the crude oil consisted of: 66.21 wt. % saturates, 25.78 wt. % aromatics, 7.69 wt. % resins and 0.32 wt. % asphaltenes. The crude oil was obtained from a field in the Norwegian Sea, and was filtered twice through a 5 µm Millipore filter under vacuum in order to remove impurities. 

### 2.4. Cellulose Nanocrystals

Nanocellulose was purchased from the University of Maine. The material was manufactured at the USDA Forest Products Laboratory in Madison, WI, USA (U.S. Dep. of Agriculture). The cellulose nanocrystals were produced using 64% sulphuric acid to hydrolyze amorphous regions of cellulose material, resulting in acid resistant crystals [31]. The acid hydrolysis procedure also results in formation of surface sulphate groups on the CNC crystals. The sulphate charge density is 0.3 mmol/g [30]. The stock-dispersion had a concentration of 12 wt. %.

## 3. Experimental Methods

### 3.1. Characterization of Cellulose Nanocrystals (CNC)

#### 3.1.1. High-Temperature Aging of CNC

CNC dispersion was diluted to samples of 0.49, 1 and 2 wt. % containing 0.1 wt. % NaCl, and 40 mL of the sample was transferred to 50 mL Schott bottles with heat-resistant caps. Schott bottles containing CNC suspension were refrigerated both before and after heat aging. Heat aging was performed in a heating bath (Julabo Gmbh, Seebach, Germany) containing oil at 120 °C for aging durations of 2 h to 16.5 weeks. These trials were conducted in an identical manner to a previous study by Molnes et al. [18], except for the sample amount during aging.

#### 3.1.2. Rheology Measurements and Shear Rate

Rheology measurements were performed using a Physica MCR 301 rotational rheometer from Anton Paar (Graz, Austria). A 40 mm diameter cone-and-plate geometry was used with roughened surfaces and a cone angle of 2°. The truncation gap size was 0.057 mm. All rheology measurements were carried out at 20 °C. The rheometric protocol for all samples comprised of the following stages: (1) a 60 second pre-shear (100 s^−1^) stage, (2) 60 s period of quiescent rest, (3) two consecutive cycles of increasing and decreasing shear rate from 0.1–1000 s^−1^. Each step in the protocol lasted for 600 s, and the duration of the entire protocol was 42 min. The concentration of samples was not altered before rheology measurements and each analysis was performed at least twice. For all comparison of viscosity data, viscosity values were extracted from a shear rate of 1 s^−1^.

The shear rate withinthe porous media was calculated using Equation (3) [3]. The calculations were performed to relate the shear rate inside the core to relevant shear viscosity measurements.

(3) $$\gamma =\frac{4\;q\;}{A\sqrt{8\;k\;\varphi }}$$

In Equation (3), *q* denotes the volumetric flow rate (m^3^/s), *A* is the cross-sectional area of the porous media (m^2^), *k* is the single phase permeability (m^2^) and φ is the porosity. 

#### 3.1.3. Atomic Force Microscope (AFM) Pictures

Atomic force microscopy (AFM) images were obtained using a Veeco diMultimode V instrument from Veeco Instruments Inc. (Santa Barbara, CA, USA). ScanAsyst mode was employed and Bruker AFM probes with silicon tips on nitride levers were used. The scanning probes had a spring constant of 0.4 N/m. 2 × 2 μm area images were obtained of effluent samples and 5 × 5 μm area images were obtained of aged CNC samples. Nanoscope Analysis v 1.50 software (Bruker Inc. Santa Barbara, CA, USA) was used to analyze the images. The CNC and effluent samples were prepared according to Lahiji et al. [32] Mica discs of 10 mm width were cleaved and a drop of suspension was deposited. The surface was immediately rinsed with DIW, and compressed N_2_ gas was used to dry the sample. Aged CNC samples were diluted with DIW to 0.0125 wt. % prior to deposition onto the mica. Effluent samples from the core flood were sonicated for 2–4 min and diluted 10 times in DIW.

#### 3.1.4. Interfacial Tension 

The interfacial tension (IFT) between crude oil and brine/nanofluid was measured with the pendant drop method using the drop shape analyzer DSA 100S (Kruss GmbH, 22453 Hamburg, Germany). The experimental procedure consisted of filling a glass container with the respective fluid (i.e., brine or nanocellulose) and a J-shaped needle was then immersed in the fluid and an oil droplet was created. The diameter of the needle was 1.047 mm. The drop shape analyzer DSA 100S uses the software ADVANCE to measure and analyze the droplet shape. Interfacial tension was measured at five minute intervals for 12 h, and recorded values were automatically logged in ADVANCE. The experiments were performed at ambient temperature and pressure conditions. Four measurement replicates were performed with crude oil and brine, while two replicates were performed with crude oil and nanofluid.

### 3.2. Core Plug Setup and Cleaning 

The core was mounted in a hydrostatic core holder with a Viton^®^ rubber sleeve (Elastomer Engineering Limited, Lymm, UK). The core was pressure tested at 60 bar for 16 h using nitrogen (N_2_) gas, to ensure that no leakage into the core occurred from outside the sleeve. The nitrogen gas was subsequently replaced with high-viscous paraffin oil to maintain a constant sleeve pressure during the experiment. All valves were leak tested prior to initiating the experiment. 

The Bentheimer core plug had not been used in previous experiments and was not contaminated with oil residue. Therefore, a mild cleaning procedure was applied. After the core plug was installed in the core holder inside the heating cabinet, it was rinsed by injecting methanol overnight. A low flow rate of 0.01 mL/min was used and the back pressure was maintained at 15 bar. The cleaning procedure was performed at room temperature. 

### 3.3. Establish Initial Saturation

Saturation of the core was performed using an injection scheme in which the core was mounted in the core holder on the rig. The cleaned core was first 100% saturated with brine by injecting 0.1 wt. % NaCl. This brine composition was selected for all experimental stages to avoid concentration gradients and associated uncertainties in local salinity values. The oil saturation protocol consisted of four stages and the duration of each stage is listed in Table 2. A synthetic oil (isopar L) was first injected through the core at a rate of 1 mL/min, resulting in an initial water saturation (*S*_wi_) of 36%. Subsequently, a mixture of 20% isopar L and 80% high-viscous paraffin oil was injected in two steps. An initial low rate of 2 mL/min provided a *S*_wi_ value of 22%, and a subsequent high injection rate of 9 mL/min provided a final *S*_wi_ value of 15%. The original oil in place (OOIP) was quantified as 120.5 mL. To achieve an optimal displacement of the fluid front, these first three injection stages were conducted at room temperature conditions corresponding to a high oil viscosity. In the final step of the oil saturation procedure, crude oil was injected to replace the 20/80% isopar L/HVP oil. This was done at 60 °C. The core was then aged for five weeks at 60 °C.

### 3.4. Core Flood Experiment

#### 3.4.1. Experimental Setup

**Figure 2:**
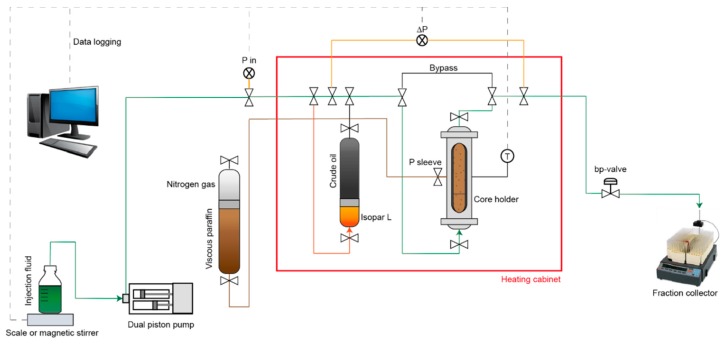
Schematic illustration of the experimental setup of the core flood.

Figure 2 illustrates a schematic of the experimental setup. The core plug was installed vertically in the core holder. Sleeve pressure was kept at 75 bar during the primary experiment using high viscous paraffin oil. A cylinder containing high viscous paraffin oil (200 mL) in the bottom and nitrogen gas on the top was placed outside the heating cabinet and was used to maintain the system at equilibrium. The back pressure was kept at 15.5 bar during the main experiment and the temperature was set to 120 °C.

The injection fluid container was placed on a balance (during water flooding) or on a magnetic stirrer (during nano flooding). The balance measurements were fed to a computer for data logging. The balance was not able to be used in combination with a stirrer, but the pumping rate was tested and confirmed prior to nano flooding. The sample changer also provided an opportunity to monitor the flow rate on an hourly basis. The injection fluid was pumped into the core using a Pharmacia P-500 pump. The differential pressure (dP) across the core was logged, as well as the inlet pressure and the temperature inside the core holder. During saturation of the core, a cylinder containing crude oil at the top and isopar L at the bottom was placed inside the heating cabinet for the final saturation step (replacing 20/80% isopar L/HVP oil with crude oil). Autoclave tube fittings and valves were used to direct fluid flow. A bypass line was installed over the core to allow fluid diversion during various stages of the experiment. For example, fluid diversion was applied to reduce the dead volume of the prior fluid during heating and prior to introducing a new fluid to the core. 

#### 3.4.2. Injection Scheme

After establishing initial water saturation and aging the core for five weeks at 60 °C, the temperature was increased to 120 °C. The core flood was initiated after the temperature stabilized at 120 °C. The first stage of the core flood was secondary recovery using low-salinity water (LSW) (0.1 wt. % NaCl). LSW was injected at a rate of 0.1 mL/min until no additional oil was produced and the pressure had stabilized. Near the end of the water flooding stage, the injection rate was increased to 0.5 mL/min for half a PV to determine if any capillary trapped oil at the core outlet could be released prior to the nano flood. Nanocellulose fluid was then injected in several stages as a tertiary oil recovery agent. First, the core was flooded at the same rate used during water flooding, 0.1 mL/min. Utilizing a flooding rate of 0.1 mL/min indicates that one PV requires approximately 24 h to complete; 24 h should be sufficient time for the nanocellulose fluid to undergo a substantial viscosity increase inside the core, which hypothetically should give rise to a different flow path than the pure LSW flood. By creating a different flow path, nanofluid may extract additional oil from the core. However, to provide complete certainty that the nanofluid was able to properly viscosify inside the core, it was decided to shut-in the core for 24 h. During the shut-in period, the content of the core was un-extracted oil and nanocellulose fluid, while the bypass line was flooded with brine to prevent the flow lines from becoming constricted with viscous nanocellulose fluid. After 24 h, nanofluid injection continued ata low rate of 0.1 mL/min. The injection continued until no additional oil was produced. Subsequently the rate was increased in three stages to 0.3, 0.5 and 1.0 mL/min, respectively. It was decided to increase the rate in steps, as there was uncertainty regarding how shear thinning the nanofluid was. The pressure gauge could only read values up to five bar before it saturated. Therefore, it was important to build the pressure up gradually so that it was maintained below this value. During all nano flooding stages the goal was to inject nanofluid until no additional oil was produced and a stable pressure reading was attained. The final step of the core flood was a post flush, in which low salinity water was injected at a rate of 0.5 mL/min.

### 3.5. Permeability

Permeability was measured initially, after each core flood stage, and after rinsing the core. It was measured by flooding with the respective fluid at four different injection rates until a stable differential pressure (dP) was obtained, and then it was calculated using the Darcy equation.

### 3.6. Effluent Characterization

Effluent samples were collected at all times during the different stages of the core flood.

#### 3.6.1. Oil Production

Oil produced during core flooding was measured in one of two ways, either from vials from the fraction collector or by graded measuring cylinders. The vials in the fraction collector were not graded; the calibration line was established based on reading the height of volume in centimeters and measuring the weight for each reading. The volume was plotted against the height for all the reference samples and a factor was established to correlate fluid height (cm) directly to volume (mL).

For the graded cylinders, it was difficult to estimate the amount of oil when reaching the tail of the production for each flood. A weight-based method was implemented to obtain a more accurate oil volume estimation. Toluene was added to the measurement cylinder containing oil and water and the toluene/oil phase was extracted using a syringe. The mixture was then added to an empty beaker of known weight, and re-weighed. The beaker was subsequently placed over a heating bath (60 °C) for some hours and then placed in an oven (80 °C) so that the toluene could evaporate over time. The beaker was weighed over three days until it stabilized. The amount of crude oil in the beaker was then scaled using a factor that was determined from a reference set. The reference set (three samples) consisted of a known amount of oil and toluene added to a beaker and reading the final weight at the end after evaporation. The difference in weight from start to end was calculated and based on a factor that was determined on how much oil was lost to evaporation.

#### 3.6.2. Particle Size

Particle size measurements were performed on selected effluent samples. These effluent samples were collected during the nano flood and the idea was to investigate the size distribution throughout that stage to see if it could yield additional information concerning the processes occurring inside the core. Particle sizes were, therefore, measured prior to oil breakthrough, at oil breakthrough, at the end of first low rate stage, after shut-in, at the end of the second low rate (after shut in), and at two high rate stages (0.5 mL/min and 1.0 mL/min). The particle size was measured at room temperature using a Zetasizer Nano ZS instrument (Malvern Instruments Nordic AB, Skallestad, Norway).

### 3.7. Characterization of the Core after Core Flood 

#### 3.7.1. Micro Computed Tomography (Micro-CT) Scan

**Figure A1:**
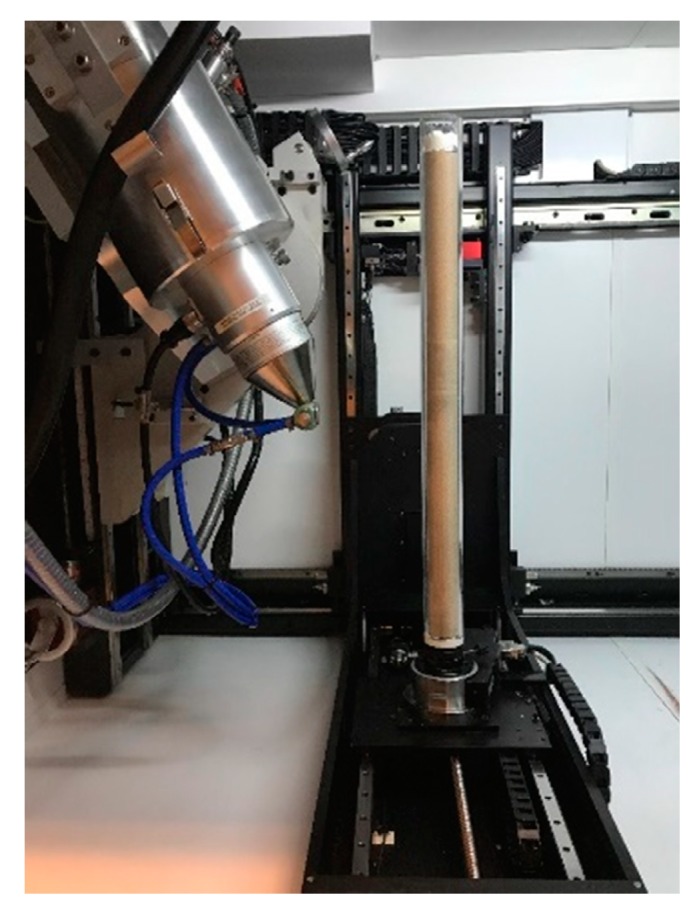
Inside view of micro computed tomography (micro-CT) scanner. On the left is the x-ray source. The sample is sitting on the rotating stage. After one sub scan, the sample will be moved one step up for another sub scan.

A micro computed tomography (micro-CT) scan was performed to determine if the core was homogenous or if high permeable flow paths were present in the core. The micro-CT scan was performed on a Nikon XT H225 scanner (Nikon Metrology, Tring, UK). The scan was executed after the core flood was completed and the core had been rinsed and dried. The core was placed in a plastic holder (Figure A1, Appendix A), and due to the core length the equipment could only scan half of the core at a time. One scan (half the core) consisted of seven sub-scans at 205 kV and 110 µA with exposure time 0.14 s. The image resolution is 27.4 µm/voxel. After reconstruction, one image stack was generated for each sub-scan. All image stacks were subsequently stitched together using the program ImageJ.

**Figure A2:**
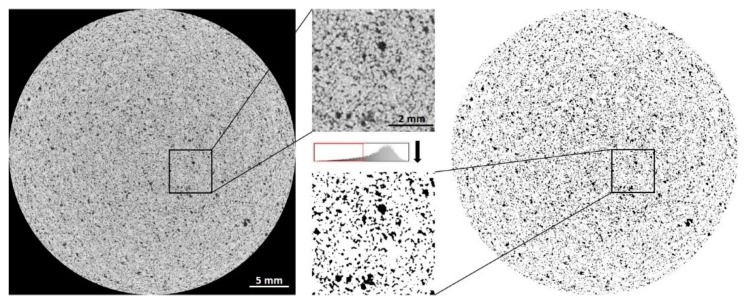
On the left is one micro-CT slice. The black area around the image is air. The grey value represents matrix in the sample. The black area in the slice represents pore space. The top image in the middle is one zoom in area indicated with a black square in micro-CT slice, to give better visualization of sample components. The histogram of this micro-CT slice is in the middle. The red rectangle indicates the grey value range of pore space. The bottom image in the middle is the segmented image when the threshold value applied. The complete segmented result of this micro-CT slice is given on the right.

The image segmentation was based on the range of the grey value, i.e., black represents pores, and grey to light grey represents matrix, e.g., quartz. Single threshold method was applied between pores and matrix (Figure A2, Appendix A). The threshold value was based on visual observation. Therefore, if there were any pores smaller than the voxel size they would not be captured via the micro-CT technique.

#### 3.7.2. Scanning Electron Microscopy (SEM) Imaging

**Figure 3:**
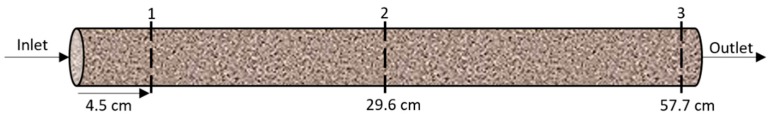
Illustration of Bentheimer core. The core was cut in three places where each piece was 1.5 cm thick. These three pieces were investigated for nanoparticles using scanning electron microscopy (SEM) imaging.

Scanning electron microscope (SEM) images were acquired of selected areas of the core to determine how far the nanoparticles were stuck inside the porous media. The core was cut in three places in total where each piece was 1.5 cm thick (Figure 3).

The three core pieces were coated with gold and investigated using SEM system from FEI (Field Electron and Ion) company (model Apreo) in the Norwegian University of Science and Technology (NTNU) NanoLab. The same SEM acquisition setup was applied on one clean unused Bentheimer core. The image of this core sample was used as reference for comparison with the images of the cut core pieces from the flooding experiment.

## 4. Results and Discussion

### 4.1. Characterization of CNC

#### 4.1.1. High-Temperature Aging of CNC

**Figure 4:**
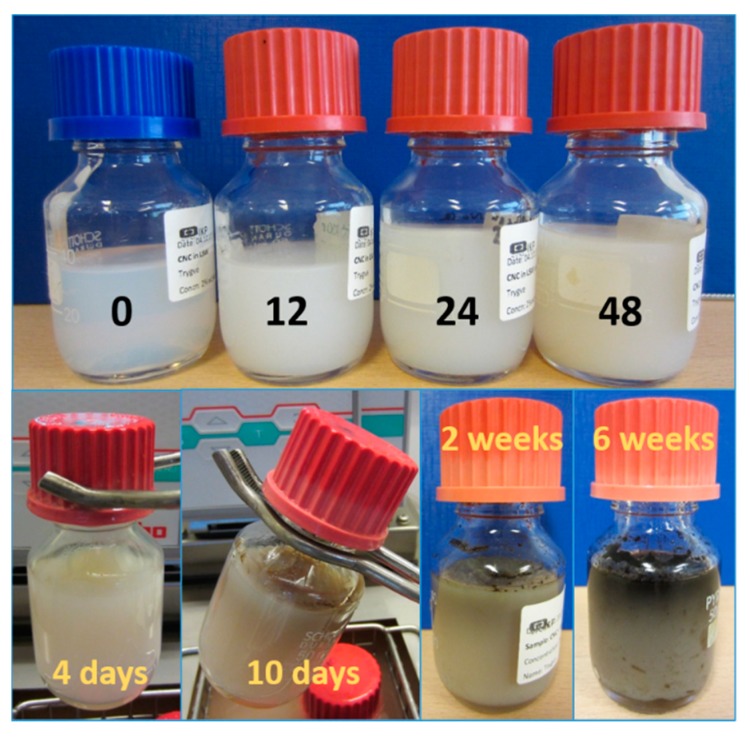
Pictures of 2 wt. % cellulose nanocrystals (CNC) in low-salinity water (LSW) after aging at 120 °C for different periods of time. The top row shows samples after aging times of 0, 12, 24 and 48 h. The bottom row shows samples after 4 days to 6 weeks.

After aging at 120 °C, changes in the nature of the CNC suspension were clearly visible, as displayed in Figure 4. While the unaged sample was slightly transparent, all aged samples were translucent (i.e., not transparent). After 12 h, the suspension had attained a gel form able to sustain its shape against gravity. The CNC suspension became altered from a pale white color at 12 h to increasingly beige hues after each subsequent day. After 10 days, black and grey tones, indicative of charring, became visible, and after 6 weeks, the top half of the sample was completely black, Undoubtedly, oxygen gas trapped above the suspension played an essential role in the charring process, but most likely does not contribute to the observed viscosity increase.

The color change followed the same color scheme observed by Molnes et al. [18], but over dissimilar time scales. There, the samples were completely black after one week, while 6 weeks of aging time were required before true black areas were observed. The dissimilar time scales are believed to be due to differing amounts of sample in the bottles, as this is the only known difference between the samples of the current study and the samples from the Molnes et al. [18] study. The current study used approximately three times as much sample in containers of identical size. Therefore, after the viscosity increased and the rate of diffusion diminished, a smaller volume was accessible to chemically reactive species. This hypothesis is based largely on the color gradient observable across the sample height.

**Figure 5:**
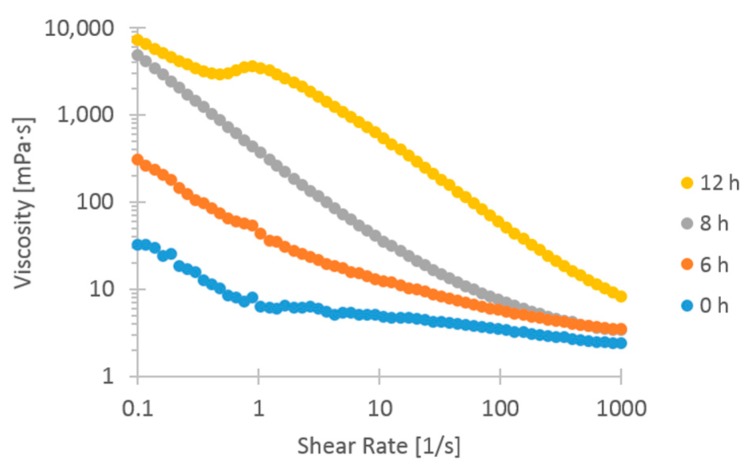
Shear viscosity curves of 2 wt. % CNC aged at 120 °C up to 12 h. The displayed viscosity values were measured at shear rate 1 s^−1^ from the first cycle of decreasing shear rate.

**Figure 6:**
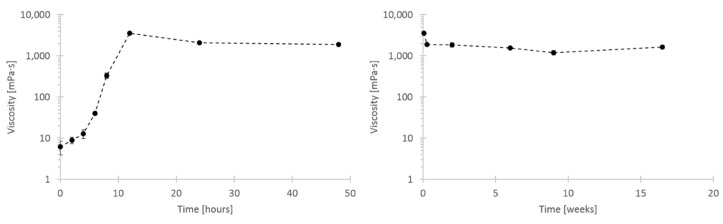
Shear viscosity of 2 wt. % CNC aged at 120 °C in static containers. The displayed viscosity values were measured at shear rate 1 s^−1^ from the first cycle of decreasing shear rate.

Results from Molnes et al. [18] show drastically increased viscosity of CNC dispersions after 24 h of heat aging at 120 °C. These results are repeated and expanded upon in the current study. Figure 5 shows these viscosity results for 2 wt. % samples of CNC, demonstrating that the viscosity increase begun immediately and Figure 6 shows that the viscosity reached a maximum after about 12 h, after which the viscosity was stable for over 16 weeks. The heat aging study was subsequently terminated, without observing any viscosity reduction. Heggset et al. [30] found that during high-temperature heat aging of CNC and CNF a form of thermo-oxidative degradation called oxidative-reductive depolymerization, takes place, which is propagated via hydroxyl radicals.

The persistence over time of this high viscosity state is highly advantageous for oil reservoir applications as residence time in the reservoir may be in the order of many months.

**Figure 7:**
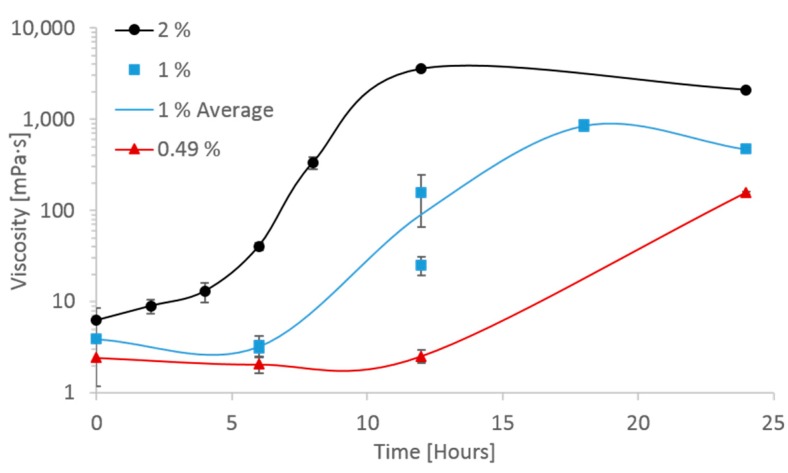
Shear viscosity at shear rate 1 s^−1^ of CNC aged at 120 °C, from the first cycle of decreasing shear rate. The concentrations of CNC are given in wt. %. Aging of 1 wt. % samples at 6, 12 and 18 h was conducted twice. Error bars signify standard deviation between shear viscosity replicate measurements. The other aging tests were conducted only once. The 1% line follows the average between test replicates.

The shear viscosity values and shape of curves fit well with the results of Molnes et al. [18], but the onset of the viscosity increase at static conditions has not previously been studied. The long-term stability of increased viscosity is also elucidated in the current study. The aforementioned study had suggested some difference in onset and rate of viscosity increase depending on initial CNC concentration of the suspension. Due to the importance of selecting a representative CNC concentration in the current study, corresponding to relevant core flooding parameters, the concentration dependence of the viscosification phenomenon was also studied. The results are shown in Figure 7, and proved vital for understanding the viscosification time scale.

As shown in Figure 7, the 2 wt. % CNC sample reached a maximum viscosity after approximately 12 h of aging time, but the 0.49 wt. % sample showed no sign of change at 12 h. All three concentrations underwent a substantial viscosity increase after 24 h, with higher viscosity as a function of higher concentration. The extreme effect of heat aging on the 2 wt. % CNC samples, manifested by both the immediate onset of viscosity increase and the magnitude of the viscosity increase (three orders of magnitude) gave cause for concern that the nanofluid was likely to plug the core completely, with the consequence of shutting down the experiment before results could be obtained. The late onset of viscosity increase for the 0.49 wt. % CNC samples were also deemed problematic, as it is desirable to maintain elevated viscosity through a core section of maximum length to mobilize the greatest amount of trapped oil. A compromise of 1 wt. % was chosen, with the hope of avoiding both the extremes of plugging the core, and CNC travelling through the core without having aged. 

#### 4.1.2. Interfacial Tension

The interfacial tension between crude oil and brine/nanofluid was measured at ambient pressure conditions using the pendant drop method. The experiments were performed at room temperature, as the equipment did not have the necessary components to perform measurements at 120 °C. The results (see Appendix B), demonstrate that the nanofluid exhibited a reduced interfacial tension in comparison to low salinity water. At the start of the experiment, the IFT for CNC was 1.4 mN/m lower than the reference sample and after 12 h the difference was 2.3 mN/m for the two systems. In other words, the interfacial tension reduction is modest. Thus, IFT reduction is, therefore, likely not a dominant mechanism for incremental oil production.

### 4.2. Oil-Recovery Experiment

**Figure 8:**
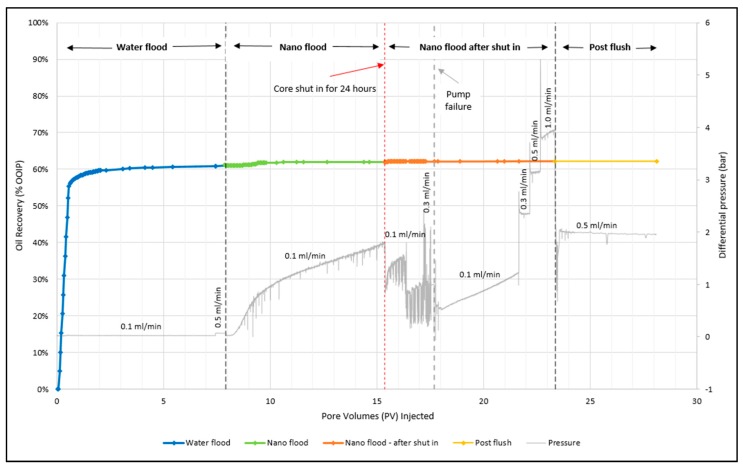
Oil recovery in % OOIP of low salinity water flood (blue line), nano flood (green and orange line) and post flush (yellow line) as a function of injected pore volumes (time). The grey line is the corresponding pressure curves. The experiment was conducted at 120 °C.

Experimental results from the core flood are presented in Figure 8 and Table 3. The core flood was conducted at 120 °C with an initial water saturation (*S*_wi_) of 15%. The core was aged for five weeks using crude oil. The study was performed in three flooding stages. First, low salinity water (LSW) was injected to recover as much oil as possible, then nanocellulose was injected as an enhanced oil recovery method to determine if incremental oil could be produced. Finally, a post flush with LSW was conducted. The core flood methodology was to continue each stage until no additional oil was produced and the pressure had stabilized. For the water flood 7.4 PV was injected at a low rate of 0.1 mL/min, which resulted in an oil recovery of 60.8%. The rate was then increased to 0.5 mL/min and an additional 0.21% oil was produced. This might be capillary trapped oil that was released from the outlet. Figure 8, shows that the pressure stabilized for both water flood stages.

The nano flood scheme consisted of several stages. CNCs were first injected at a similar low rate as LSW flooding, until no additional oil was produced. From this stage, it was observed that the pressure was considerably higher than for water flooding. By comparing the pressure at the end of water flooding to the end of nano flooding, the pressure was 65.1 times higher for a nano flood. Furthermore, the pressure also kept increasing and did not stabilize. However, the last oil was produced around 14.4 PV and nanofluid injection continued until 15.4 PV was reached. Although the differential pressure continued to increase, the increased pressure differential did not give rise to additional oil production. Merely 0.92% incremental oil was recovered during this stage. To ensure that the nanocellulose had sufficient time to viscosify inside the core, the core was shut in for 24 h (this event is denoted by the red dotted line in Figure 8). After 24 h, CNC was injected through the core at a low rate of 0.1 mL/min, and an incremental oil recovery of 0.14% was achieved. During this stage two PVs were injected, even though oil was produced only during the first 0.21 PVs. The pressure was spiky during this stage, and this might be attributable to log-jams building up and breaking free. Log-jam formations occurs when particles accumulate at pore throats, causing pore constrictions and increased differential pressure. Log-jams are a form for mechanical entrapment of particles [20]. At approximately 16.4 PV, the pressure dropped somewhat, which might be caused by a main flow path reopening after being constricted by nanocellulose agglomerates. The pressure never stabilized, but no additional oil was produced so it was decided to proceed with the next flooding stage.

High-rate nano flooding was performed in three stages in order to prevent that the pressure gauge was capped. In the first attempt for the high rate (0.3 mL/min) the pump malfunctioned at 17.7 PV, and the core was shut in while repairing the pump. The pump was quickly repaired, but it was decided to inject additional PV using the low rate (0.1 mL/min) to ensure proper functioning of the pump. The low rate was continued until 21.7 PV was reached and during this stage 0.09% incremental oil was produced. The pressure kept increasing and never stabilized as was previously observed for the other nano flood stages, but with no large spikes of pressure as was seen before. High-rate injection of 0.3 mL/min was then re-initiated and 0.02% incremental oil was produced. At this rate, as well as for the second high rate of 0.5 mL/min, the pressure seemed to be stable. However, the second high rate did not produce any additional oil. Finally, a high injection rate of 1.0 mL/min was applied and continued until 23.4 PV was reached. The pressure increased slightly during this stage, but no extra oil was produced. Overall, 15.5 PV of nanofluid was injected during the nano flood stage, and it resulted in a total oil recovery of 62.2%. This means that the nanofluid contributed to an extra 1.2% oil being recovered after LSW flooding. 

At the end of the water- and nano-flood stage permeability measurements were conducted using four different flow rates (at 120 ° C). The effective permeability near the end of the water flooding stage was calculated to be 205.3 mD (where µ_w_ = 0.233 cP, Table 1), while the effective permeability near the end of the nano flooding stage was calculated to be 753.9 mD. In order to take into account that the CNC had been aged for a certain time inside the core during the experiment, the viscosity of the nanofluid was calculated according to the kinetics in Figure 7. Because CNC is a non-Newtonian fluid, the calculations were based on the viscosity after 10 h, providing a reasonable estimate for the average CNC viscosity inside the core during the experiment (µ_n_ = 30 cP, from Figure 7). The effective permeability of oil had been calculated during the oil saturation stage of the core and was 1965.3 mD (where µ_o_ = 3.32 cP, Table 1). From these values, the mobility ratio for water- and the nano flood could be calculated using Equation (2), and was 1.49 and 0.04, respectively. Thus, nano flood should give a favorable mobility ratio, which could indicate that the fluid behaved more like a viscous polymer front compared to water.

The post-flush stage was performed as a final step in which LSW was injected at 0.5 mL/min for 4.8 PV. With this stage, the idea was that the LSW could help break-up nanocellulose agglomerates and push the nanoparticles out from the core. In addition, if the nanocellulose had released some additional oil, but was unable to transport it all the way out due to the agglomerates, perhaps LSW could help facilitate this oil movement. Injection was continued until the pressure stabilized. However, no additional oil was produced during this stage.

A recovery factor of 61% during water flooding is a high recovery factor. Thus, it is challenging to try to achieve a higher recovery factor after this stage. Generally, polymers can provide excellent incremental oil recovery factors only when the recovery factor of the initial water flooding stage is poor (0–40%) [33,34]. In the current core flood, water flooding appears to have provided excellent sweep efficiency of the core plug. Thus, the remaining oil proves difficult to extract using a high-viscosity aqueous front (i.e., nanocellulose fluid). 

### 4.3. Permeability

At 100% water saturation, the core permeability was measured to be 1980 mD. After the nanofluid core flood experiment, the core was rinsed with methanol and toluene in alternate sequences. The permeability was then measured using methanol and was calculated to be 192.4 mD. The substantial permeability reduction provides strong evidence of nanoparticle retention inside the core. Reverse flooding was therefore implemented to extract large nanoparticle agglomerates that are unable to transverse the core. Such large agglomerates would be immobilized near the core inlet, and reverse flooding was implemented to remove the nanoparticle agglomerates from the core inlet and recover the initial core permeability. After reverse flooding was conducted, the permeability was tested in the reverse direction using methanol yielding a permeability value of 207 mD. This was a small increase in permeability of 14 mD, but still represents a substantial deterioration from the initial core permeability. Thus, the porous media suffers a significant permeability impairment during nano flooding. 

**Figure A4:**
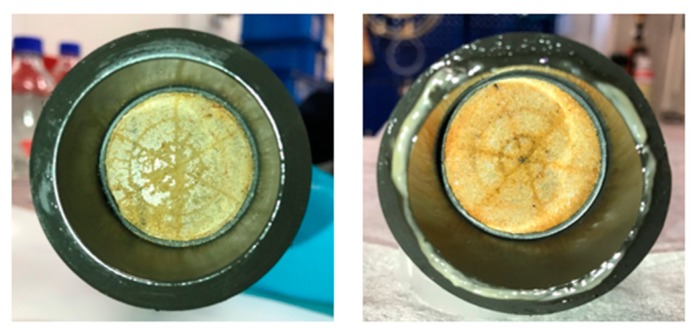
Left side: Inlet with thin nanocellulose filter cake. Right side: Outlet with no visible nanocellulose layer on it.

The core was dismantled after the flood, and the inlet side and outlet side were further investigated. By visually observing the inlet side one could see a thin white nanocellulose layer, functioning as a filter cake (Figure A4, Appendix C). From the outlet side it was not possible to observe any visible nanocellulose layer. 

### 4.4. Characterization of the Core after the Core Flood

The micro-CT scan shows that the core plug was quite homogenous (see Appendix C, Section C.2). 

#### SEM Imaging

**Figure 9:**
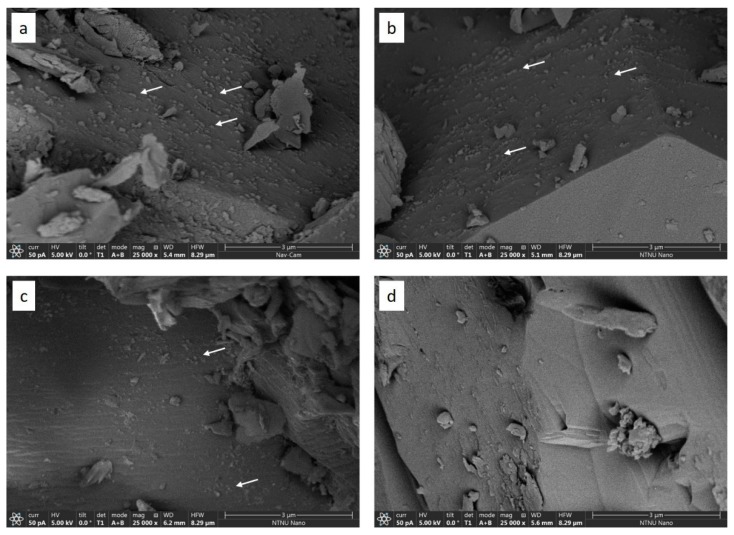
Image **a**, **b**, and **c**: SEM image of piece 1, 2, and 3 with resolution 5.4 nm. White arrows pointed out some of the nanoparticles in pore space. Image **d**: SEM image of clean reference core with resolution 5.4 nm/pixel. There is no similar particles in pore space showing in the image.

In Figure 9, there are four SEM images with resolution 5.4 nm/pixel. Images of a, b, c in Figure 9 correspond to the pieces of 1, 2, and 3 of core cuttings (Figure 3). Image d is from clean core without nanoparticles. The SEM images focus on the pore space in the core pieces. The white arrows in image a, b, and c point out some of the particles which cannot be seen in image d. Because the nanoparticles can be seen in both piece 1 (close to inlet) and piece 3 (close to outlet), it indicated that the nanoparticles traversed the entire core, and were immobilized in all core regions, even though a filter cake was formed at the inlet side (Appendix C, Figure A4). From the images it was observed that there were more nanoparticles at the inlet side than the outlet side. However, the amount cannot be determined quantitatively using this method, as this is a qualitative approach. The observation of nanoparticles present within the core, even after rinsing with toluene and methanol, supports the hypothesis of the effluent concentration being significantly lower than the injection fluid concentration. However, selective filtration of CNC particles based on particle size is more difficult to ascertain based on the SEM images. In comparison to the CNCs observed in AFM images in Figure A7 (see Appendix D), the nanoparticles in the SEM images seemed to be of roughly the same length range. However, the characteristic elongated shape of CNC particles was not seen.

### 4.5. Effluent Characterization 

#### 4.5.1. Particle Size

CNC particles are very anisotropic as seen in AFM images (Figure A7, Appendix D). Dynamic light scattering (DLS) allows determination of an average diameter of a sphere with the same diffusion coefficient as the CNC particles.

**Figure A6:**
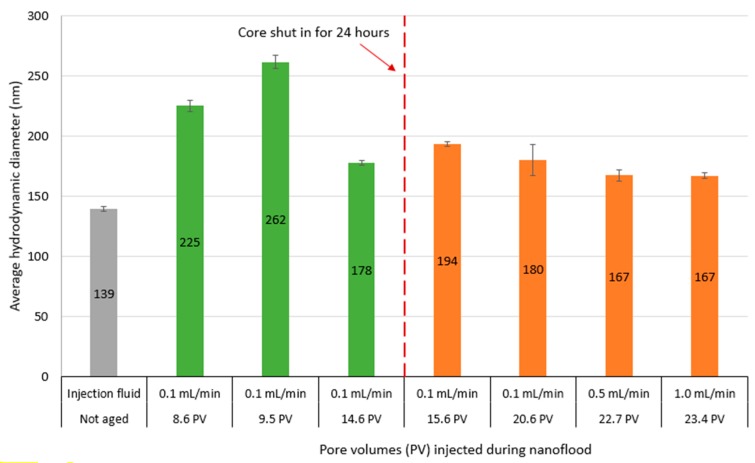
Particle size at different stages during the nano flood. The red dotted line illustrates the time when the core was shut in for 24 h. The green and orange color corresponds to the colored lines in the nano flood in Figure 9.

Based on the particle size measurements, it was established that the injection fluid had an average particle diameter of 139 ± 2.0 nm (Figure A6, Appendix D). Throughout the nano flood effluent samples were collected and the particle size was measured on selected samples to see how it changed during the flood. The first particle size measurement was taken at 8.6 PV, which was before any oil had been produced during the nano flood stage. The effluent particle size was slightly larger (225 ± 4.4 nm) than what was measured for the unaged injection fluid. The apparent disparity in particle size indicates that a change in particle characteristics occurs in the porous media, because of increased temperature, particle agglomeration, and/or size-selective filtration processes. At 9.5 PV, oil was produced from the core and the particle size reached its maximum value during the nano flood, 262 ± 5.5 nm. In other words, it seemed like the particles were slightly increasing in size until oil was being produced. At 14.6 PV, the production of oil had stopped, and the particle size was reduced to 178 ± 2 nm, but it was still higher than the initial size. After the core had been shut in for 24 h, the nanocellulose fluid inside the core should have increased in viscosity. From the particle size measurement afterward, an increase in particle size was observed compared to the value before shut in. However, the particle size after shut-in was not as high as it was during the oil breakthrough. One measurement was also performed towards the end of the second low rate after shut in (20.6 PV), and the size was approximately the same as immediately after shut in, 180 ± 13 nm. For the two high rates at the end of the nano flood, the size seemed to have stabilized around 167 nm, these were also the smallest sizes that were observed during the nano flood.

The particle size observations collectively suggest that the particle agglomeration occurs in the initial stages of the nano flood, as evidenced by the increased size for the two first measurements. Initially, all the flow paths that the water has flown through should in principle also be open for the nanofluid. This reasoning suggests that larger agglomerates of particles have the ability to traverse the core, as the biggest pore throats should be open. Over time, as additional particles traverse the core, they could start to build up at the pore throat, creating a log-jam. A log-jam means that two or more particles with sizes slightly smaller than a pore throat arrive at the pore throat together, blocking the path, thus making it hard for large particle agglomerates to traverse the core. Over time, upon continuous nanofluid injection, the particles will continue to block off pores and pore throats in the core. It is believed that this occurs inside this core, and this is further supported by the pressure data in Figure 8. During the different stages of the nano flood the pressure kept increasing, which is an indication that either oil is being mobilized inside the core, and/or pores are being blocked off. After the big oil production in the beginning of the nano flood, there appear to be a filtering of large particles inside the core, as differential pressure continues to increase and particles exiting the core are smaller in size. During the final stage of the nano flood, the particle size seemed to be relatively constant and the pressure was also more stable. These observations confirm that the porous media was filtering the large particles, but not blocking off more pore throats, and there were some flow paths open where the small sized particles could traverse the core.

Formation of log-jams in high permeable channels may divert the fluid into un-swept low permeable channels, however a premise for fluid diversion is that the subsequent fluid contains particles that are small enough to be able to pass through the narrower pore throats. This could be a mechanism of producing more oil, as the fluid would be introduced to new flow paths inside the core. However, if the particles are large (which they seem to be in this case), the fluid will not have the possibility to sweep the narrower flow paths. Therefore, the idea behind the post flush after the nano flood was that it would potentially sweep a new area of the core. Nevertheless, it seemed like the nanocellulose fluid had blocked off most of the core at that point, because there was no extra oil being produced during that stage and the core had a permanently impaired permeability.

#### 4.5.2. Rheology Measurements

**Figure 10:**
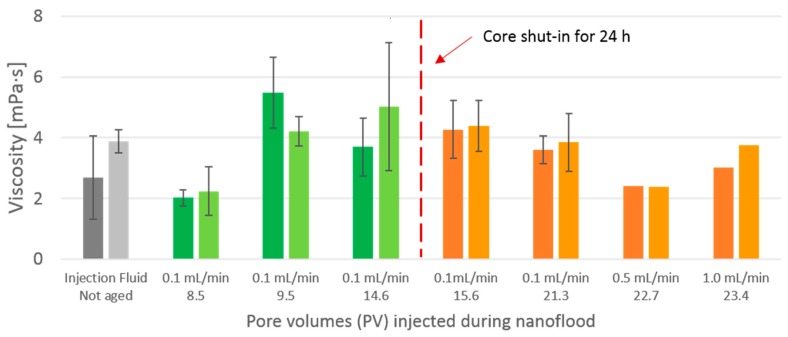
Shear viscosity at shear rate 1 s^−1^ of injection fluid and core flood effluent. The dark bars are from increasing shear rate curves and light bars are from decreasing shear rate curves.

Shear viscosity at low shear rate was investigated for core flood effluent from different stages of the core flood. The results are displayed in Figure 10. Effluent samples were acquired from the same flooding stages as those displayed in Figure A6 (Appendix D), but slightly different times. As each hour of flooding only produced 6 mL of effluent, only 2–3 measurement replicates could be measured for each sample. The two high-rate sample data points are missing error bars because one test replicate was discarded from each of them due to unreasonably high viscosity values, perhaps because of particulate matter in the sample.

The magnitude of viscosity differences observed here were quite small, which stands in stark contrast to the evolution of viscosity observed for 2 wt. % CNC during static aging. All the effluent viscosity values were in the same order of magnitude as the injection fluid. Standard deviations were substantial in relation to the difference between samples. The only effluent sample to show a distinct deviation was the sample extracted after the injection of 8.5 PV. This sample was extracted in the beginning of the nano flood, and before additional oil production was observed and had slightly lower viscosity than both injection fluid and other effluent samples. From Figure A6 (Appendix D), a higher particle size can be recorded, so aggregation, i.e., reduction of number of particles, might be the explanation. Dilution from the preexisting water in the core after water flood might also have resulted in lower concentration, causing viscosity to be lower. As explained in Section 4.3, a large reduction of permeability was observed after the core flooding was finished, and retention of particles inside the pores was assumed to be the cause. Retention of particles inside the core would lead to a reduction of particle concentration in the effluent, and could partly explain the low viscosity values. The amount of nanocellulose in the core and the concentration of particles in the effluent is unknown, so a quantitative conclusion cannot be drawn on the effect of particle retention on viscosity. The similarity in viscosity between injection fluid and effluent samples was unexpected, given the evolution displayed in Figure 7, which shows an onset of viscosity increase between 6 and 12 h aging time at 120 °C, with an eventual viscosity increase of approximately a factor of 100. 

From these results, it seems that the CNC had not been aged within the core in any manner similar to the static samples aged in Schott-bottles. A plausible account for the discrepancy is that the real residence time within the reservoir is not known. If the entire pore volume of the core had been accessible to fluid flow, the residence time should be 24 h; however, much of the pore volume is most likely inaccessible, or at least not preferred flow paths. After the experiment was finished the residual oil saturation was 32%, which may be a good estimation of inaccessible pore volume, corresponding to a residence time of approximately 16 h. Figure 7 shows, however, that the 1 wt. % CNC viscosity should have increased by more than one order of magnitude after 12 h and two orders of magnitude after 18 h. Had the CNC nanofluid reacted similarly to the high-temperature conditions as the statically aged samples, higher viscosity of the effluent should have been observed, provided that the concentration of effluent was not significantly reduced due to retention of particles in the core.

An equally plausible explanation, which could be equally relevant, is that high-temperature aging of CNC flowing through a porous system, may elapse in a completely different manner than aging performed in static bulk conditions.

Regardless of the true causality of the viscosity discrepancy, the lack of viscosity increase of CNC during core flooding is important, and can be evaluated in light of Equation (2) (mobility ratio). The viscosity ratio between displacing fluid and oil has been calculated and is displayed in Table 4. This can be used as a simplified alternative to mobility ratio, which requires additional data. 

The shear rate inside the porous media was calculated for the various injection rates used during the nano flood, see results in Table 5.

The calculated porous media shear rates were then compared against the shear viscosity measurements in order to predict what viscosity to expect inside the core. All the shear viscosity values displayed in the current study are obtained at shear rate 1 s^−1^, and viscosity values at this value are quite similar to (but slightly lower than) the calculated shear rate at the flow rate chosen for core flooding, which was 3.2 s^−1^.

Viscosity of injection fluid should be larger than viscosity of crude oil in order for the additive to be truly effective, i.e., the viscosity ratio should be larger than one. 1 wt. % CNC initially has viscosity not much higher than pure water, but during high-temperature aging the viscosity ratio increased by two orders of magnitude. The effluent did not exhibit higher viscosity as a result of the 24 h spent at 120 °C, and the viscosity ratio was not any higher than that of the injection fluid, and well below unity. 

#### 4.5.3. Atomic Force Microscopy (AFM)

**Figure A7:**
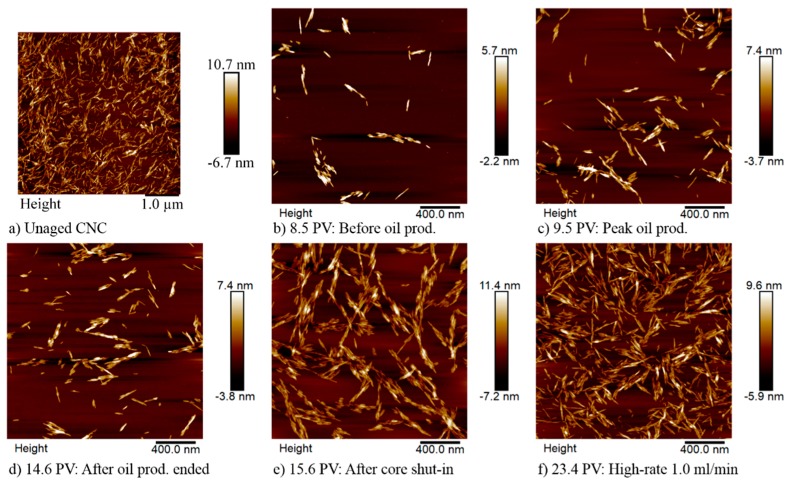
Atomic force microscope (AFM) images of injection fluid (**a**) and diluted core effluent from the nano flood stage (**b**–**f**).

AFM images were obtained of the unaged CNC injection fluid and of the effluent acquired from five different stages of the nano flood phase. The results are displayed in Figure A7, Appendix D. The images before-, at peak- and after oil production appear similar to each other, which is expected as they are subject to the same flow rate and residence time. By comparing the image with the longest residence time (after shut-in period) against the image with much lower residence time (high rate, 1.0 mL/min) there are no distinct differences between them. Thus, there does not seem to be a clear relationship between residence time of CNC in the core at high temperature and aggregation states. However, the shut-in period seem to be a marker where the effluent changes, as the images after shut in are much more similar to each other compared to the three samples obtained before shut in.

## 5. Conclusions

A high-temperature core flood experiment has been carried out in order to determine the potential of cellulose nanocrystals (CNC) as a green EOR agent for tertiary recovery. The experiment was conducted on a 60 cm Bentheimer core plug at 120 °C, and data regarding pressure and produced volumes were obtained. 

Prior to the core flood, a high-temperature aging study was performed on CNC. The study showed that the viscosity increased substantially after 24 h of heating at 120 °C, with higher viscosities obtained as a function of higher particle concentration. Based on this study it was decided to use 1 wt. % CNC in the core flood, and the flow rate was chosen to 0.1 mL/min corresponding to 24 h flooding time for 1 PV.

An initial low salinity water flood provided an oil recovery of 61%, which is considered a high recovery factor. For the nano flood, 1.2% incremental oil was recovered. During the different stages of the nano flood the differential pressure kept increasing and it was also significantly higher than during the water flood. The increasing pressure could be an indication of log-jam formation inside the core. Log-jamming may provide a mechanism for increased oil recovery and it is likely the cause of the oil production that was observed during the nano flood. Particle size measurements were also performed on the effluent nanofluid at certain times during the flood, and the results obtained correspond with the log-jamming hypothesis. In total, 15.5 PV of nanofluid were injected, which ultimately resulted in a large portion of the pore volume being constricted by agglomerated nanoparticles. This constriction was also evident from the permeability measurement after the flood, in which the permeability had decreased by 89.5% from the initial value. A thin nanocellulose filter cake was also observed at the inlet side of the core plug, which contributes to the decreased permeability. In addition, SEM images show immobilized nanoparticles throughout the core plug, with a larger portion of particles being retained at the inlet side of the core in comparison to the outlet side.

An effective EOR method should yield a minimum of 5% OOIP extra in recovery in order to be a viable candidate for an industrial operations. This means that the oil recovery in this experiment should ideally have been increased from 61 to 66%. However, since the recovery factor for the water flood is so high, it is challenging to extract the remaining oil. From the IFT measurements, a lower IFT value was observed for nanofluid in comparison to brine. Nevertheless, the IFT reduction was sufficiently small so that it would most likely not have a big effect on the overall oil recovery. For a recovery factor as high as 61%, the IFT would need to be reduced to an ultralow value (using surfactants) to be able to achieve a higher oil recovery after the water flood. In other words, in this experiment the water flood was highly successful in sweeping the core for oil. From micro-CT imaging of the core plug, it was concluded that the core was quite homogenous. It is believed that the effect of the nano flood would have been substantially improved in a heterogenic sample (layered). 

The CNC nanofluid does not seem to have increased appreciably in viscosity during flooding, as expected based on heat aging trials of CNC under static conditions. The actual nanofluid residence time within the reservoir is unknown, but even if it is half of the maximum residence time, the increase in viscosity observed under static conditions should be sufficient to provoke an increase in incremental oil recovery. It is likely that the mechanism of heat aging of CNC is very different in static conditions compared to porous flow conditions. 

The above conclusions are based on one single core flood, as it was a complex experiment and therefore not possible to do several replicates. The water flood stage contributed to a high oil recovery, which in turn resulted in a very low recovery with nanofluid. Based on this, it is difficult to conclude whether CNC may represent a good EOR candidate, as more core flood experiments should have been conducted. Future experiments should, therefore, try to implement the method earlier during water flooding, such that the recovery factor is not too high for that stage, or alternatively use a heterogeneous sample. The findings from this study can, therefore, be looked upon as preliminary results and based on the observations obtained it is believed that CNC has the potential as an EOR additive if tested under slightly different conditions. 

## Figures and Tables

**Table 1:** Fluid properties.

Fluid	Density (g/cm^3^)			Viscosity (cP)		
	20 °C	60 °C	120 °C	20 °C	60 °C	120 °C
Isopar L	0.76	0.73	—	1.33	0.72	—
20/80% isopar L/HVP oil	0.87	—	—	121.50	—	—
Crude oil	0.91	0.89	0.85	55.90	12.19	3.32
0.1 wt. % NaCl	1.00	0.98	0.94	0.91	0.47	0.23
1 wt. % CNC in 0.1 wt. % NaCl	1.00	—	—	1.40	1.09	—

**Table 2:** The different stages during saturation of the core with oil.

Stage	Fluid	Temp. Core	Flow Rate	PV Injected	OOIP	*S*_wi_
		(°C)	(mL/min)		(mL)	(%)
1	Isopar L	21	1.3	3.2	91.0	36
2	20/80% isopar L/HVP oil	21	2.1	7.0	110.4	22
3	20/80% isopar L/HVP oil	21	8.5	17.1	120.5	15
4	Crude oil	60	2.0	5.0	120.5	15

**Table 3:** Detailed overview over recovery factor (RF) from each stage and total residual oil saturation (*S*_or_).

Stage	Rate	PV Injected	Total RF	RF for Each Stage	Total *S*_or_
	(mL/min)		(%)	(%)	
Water flood (low rate)	0.1	7.4	60.80	60.80	0.33
Water flood (high rate)	0.5	0.5	61.00	0.21	0.33
Nano flood (low rate)	0.1	7.5	61.93	0.92	0.32
Nano flood (low rate, after shut-in)	0.1	2.0	62.07	0.14	0.32
Nano flood (high rate, pump fail)	0.3	0.4	62.07	0.00	0.32
Nano flood (low rate, after pump fail)	0.1	4.0	62.16	0.09	0.32
Nano flood (high rate 1)	0.3	0.5	62.19	0.02	0.32
Nano flood (high rate 2)	0.5	0.5	62.16	0.00	0.32
Nano flood (high rate 3)	1.0	0.7	62.19	0.00	0.32
Post flush	0.5	4.8	62.19	0.00	0.32

**Table 4:** Viscosity ratio between displacing fluid and crude oil (μ_w_/μ_o_) at 20 °C. Aged and unaged CNC was of concentration 1 wt. %. All values are from the first cycle of decreasing shear rate.

Flow Rate (mL/min)	Shear Rate (s^−1^)	Unaged CNC	Aged CNC		Effluent	
		0 h	18 h	24 h	Lowest visc.	Highest visc.
**0.1** **1**	3.1	0.04	6.24	5.07	0.02	0.04
	32	0.03	0.96	0.98	0.03	0.06

**Table 5:** Calculated shear rate in core at different flow rates.

Flow Rate	Shear Rate in Core
(mL/min)	(s^−1^)
0.1	3.2
0.3	9.7
0.5	16.1
1.0	32.2

**Figure A3:**
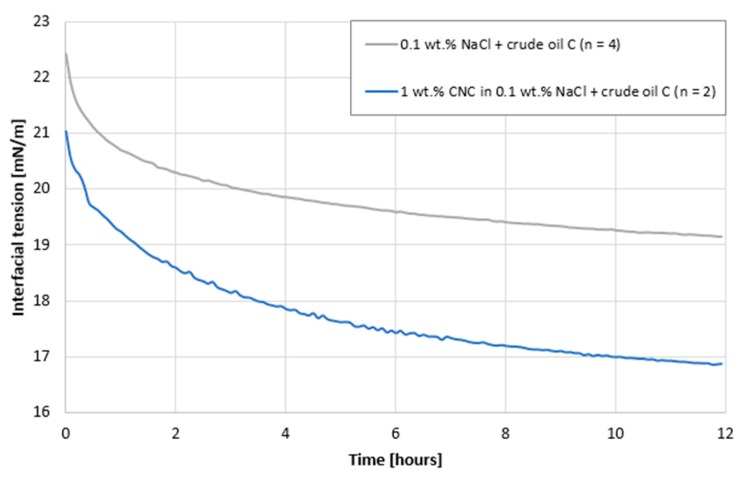
Interfacial tension over time. Grey line is the average interfacial tension (IFT) of brine and crude oil (*n* = 4), while blue line is the average IFT for nanofluid and crude oil (*n* = 2). The experiments lasted for 12 h and were performed at room temperature.

**Figure A5:**
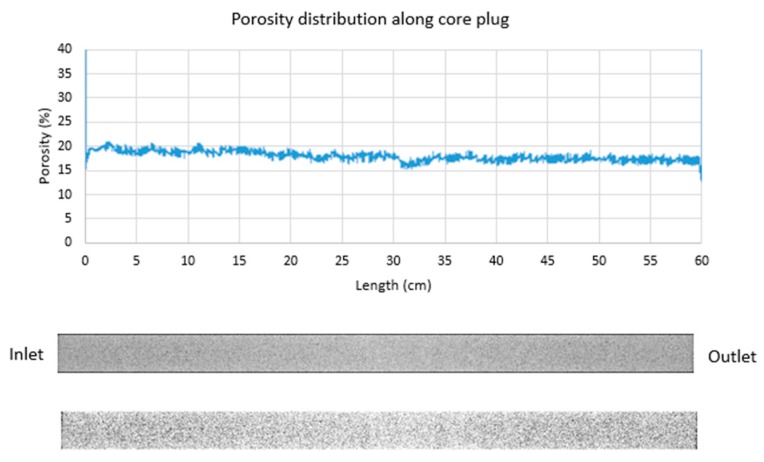
The plot above is the porosity profile through the whole core plug from inlet to outlet. In the middle is an image of the 2D (XZ) profile along the 3D micro-CT image. At the bottom is the corresponding segmented image of the profile image in the middle.

## Appendix B

### Results of Interfacial Tension Measurements

Interfacial tension between crude oil and brine (0.1 wt. % NaCl) was measured as a reference case, so that the IFT to nanofluid and oil could be compared against it. Four replicate measurements were performed for the reference sample and the average IFT of those is plotted in Figure A3 (grey line). The average droplet size was 53 ± 2.3 µL and the average temperature was 23 ± 1 °C. From the graph, it is observed that IFT did not stabilize over the duration of the experiment (12 h). These experiments will, therefore, show the general trend for IFT; the starting point, how steep the decrease was and the IFT value obtained after 12 h. For the reference sample, the IFT started at 22.4 ± 0.3 mN/m and after 12 h it was 19.2 ± 0.3 mN/m.

For the nanofluid and crude oil, two replicates were run, and the average is shown in Figure A3 (blue line). The average droplet size was 47 ± 1.7 µL and the average temperature was 26 ± 0.5 °C. The same trend was observed in this case, with a decrease in IFT over time and the value did not stabilize over 12 h. However, for this experiment IFT started at 21.0 ± 0.7 mN/m, which is a lower value than what was observed for the reference sample. After 12 h, the IFT was 16.9 ± 1.2 mN/m.

## Appendix C

### Results of Micro-CT Scan

The total porosity of the core calculated based on micro-CT image is 18.63% (Figure A5), which is 3% less than measured value in the lab. Due to the limit of micro-CT technique, any component smaller than the resolution (27.4 µm/voxel) cannot be visualized. Therefore, the porosity via micro-CT can only be used as a reference in this work. However, the distribution of porosity along the core indicates that the sample is quite homogeneous (Figure A5).

## Appendix D

### Results of AFM Images

AFM images were obtained of the unaged CNC injection fluid and of the effluent acquired from several stages of the nano flood phase. The results are displayed in Figure A7. The particle concentration difference seen between (a) and the other images is due to different methods of preparation for imaging. For images (e) and (f) it proved difficult to find sample sections with individual crystals and crystal aggregates such as seen in images (b), (c) and (d). It is unclear whether this phenomenon corresponds to an actual tendency towards aggregation.

Images (b), (c) and (d) appear similar to each other, which is expected as they are subject to the same flow rate and residence time, the only difference between the samples being to which degree the particles may have been in contact with crude oil. From these images there does not seem to be a clear relationship between residence time of CNC in the core at high temperature and aggregation states. Particles in image (e) have much longer residence time than those in (b), (c) and (d), while (f) would have had much lower residence time. Despite this, (e) and (f) are much more similar to each other than to the other three samples.
